# Colon stenosis due to acute neonatal appendicitis in a preterm baby: a case report

**DOI:** 10.1186/s12887-019-1873-0

**Published:** 2019-12-12

**Authors:** Takuto Naito, Hiromu Teramen, Hiroaki Hayashi, Mai Takegawa, Haruko Sakamoto, Toshihide Shimada, Koichi Ohno, Misao Yoshii

**Affiliations:** 1 Department of Neonatology, Osaka Red Cross Hospital, 5-30, Fudegasakicho, Tennoji Ward, Osaka, Japan; 2 Department of Pediatrics, Osaka Red Cross Hospital, 5-30, Fudegasakicho, Tennoji Ward, Osaka, Japan; 3 Department of Pediatric Surgery, Osaka Red Cross Hospital, 5-30, Fudegasakicho, Tennoji Ward, Osaka, Japan; 4 Department of Pathology, Osaka Red Cross Hospital, 5-30, Fudegasakicho, Tennoji Ward, Osaka, Japan

**Keywords:** Neonatal appendicitis, Colon stenosis, Preterm

## Abstract

**Background:**

Colon stenosis and acute appendicitis are rare diseases among premature babies. To the best of our knowledge, no study has identified both the conditions in preterm babies.

**Case presentation:**

Here we report a case of a preterm Japanese male baby who developed ascending colon stenosis and appendicitis. During his neonatal intensive care unit stay, he developed increasing apnea and vomiting with rapidly worsening abdominal distention. Contrast radiographs indicated colon stenosis. Emergent exploratory laparotomy revealed ascending colon stenosis with appendix adhesion; both the lesions were surgically resected. The pathological findings suggested that he had appendicitis several weeks prior to the surgery; the onset of colon lesion seemed later than that of appendix. The perforated appendix was covered by the ascending colon, and inflammatory reactions led to the narrowing of the intestinal lumen.

**Conclusions:**

Neonatal appendicitis and colon stenosis are both challenging for the diagnosis, and early laparotomy is necessary when these conditions are suspected.

## Background

Neonatal appendicitis (NA) is a rare disease, especially among preterm babies [1]. Patients with NA generally present with nonspecific symptoms, such as a distended abdomen, vomiting, or an increasing gastric remnant [1]. Its rarity and obscure symptoms lead to difficulties in its diagnosis.

Furthermore, controversy exists concerning the difference or relationship between necrotizing enterocolitis (NEC) in preterm babies. Notably, 50% of babies with NA are premature [2]; however, approximately 90% of NEC cases are diagnosed among preterm babies [1].

Although dozens of cases have been reported and some review articles are available, to the best of our knowledge, there is no case reported on NA complicated with colon stenosis. Here we report a case of a preterm male baby who developed ascending colon stenosis and appendicitis. Written consent to publish was obtained from the patient’s parents.

## Case presentation

A Japanese boy was born at 30 weeks of gestation by elective cesarean section to a 31-year-old gravida 1 para 0 mother. He was born with an APGAR score of 4, 8 and 8 at 1, 5, and 10 min, respectively, weighing 1,490 g. He immediately breathed spontaneously but showed chest wall retraction. He was provided with a continuous positive airway pressure (CPAP) mask and was admitted to our neonatal intensive care unit (NICU).

His first several days in the NICU were uneventful, and he could tolerate breast milk. On his 7th day of life, he presented with lethargy, bilious gastric residual, and bloody stool. His laboratory results revealed elevated C-reactive protein (CRP) (0.57 mg/dL) and white blood cell (WBC) count (10,630/μL). Chest and abdominal X-ray showed no abnormal signs. Considering the possibility of sepsis, treatment with intravenous ampicillin (200 mg/kg/day) and cefotaxime (200 mg/kg/day) was initiated. His bloody stool resolved quickly, and gastric residuals decreased and then disappeared. On his 8th day, enteral feeding was resumed. His general conditions were stable, his laboratory findings apparently improved, and we completed antibiotics treatment on his 12th day of life. He was tolerant of a feeding increase, and his growth was also stable. Ventilatory support (nasal CPAP and a high-flow nasal cannula) was required until the 15th day of his life.

**Fig. 1:**
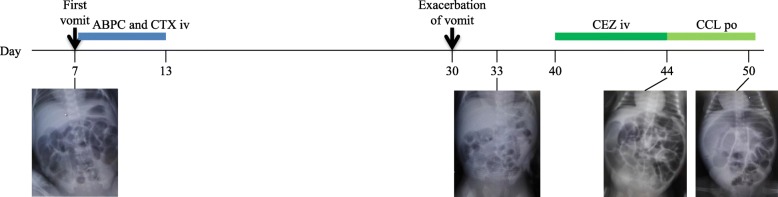
Timeline. The patient had been treated with antibiotics twice. The intestine on day 50 was distended with air. No specific evidence of air in the bowel wall was found

**Fig. 2:**
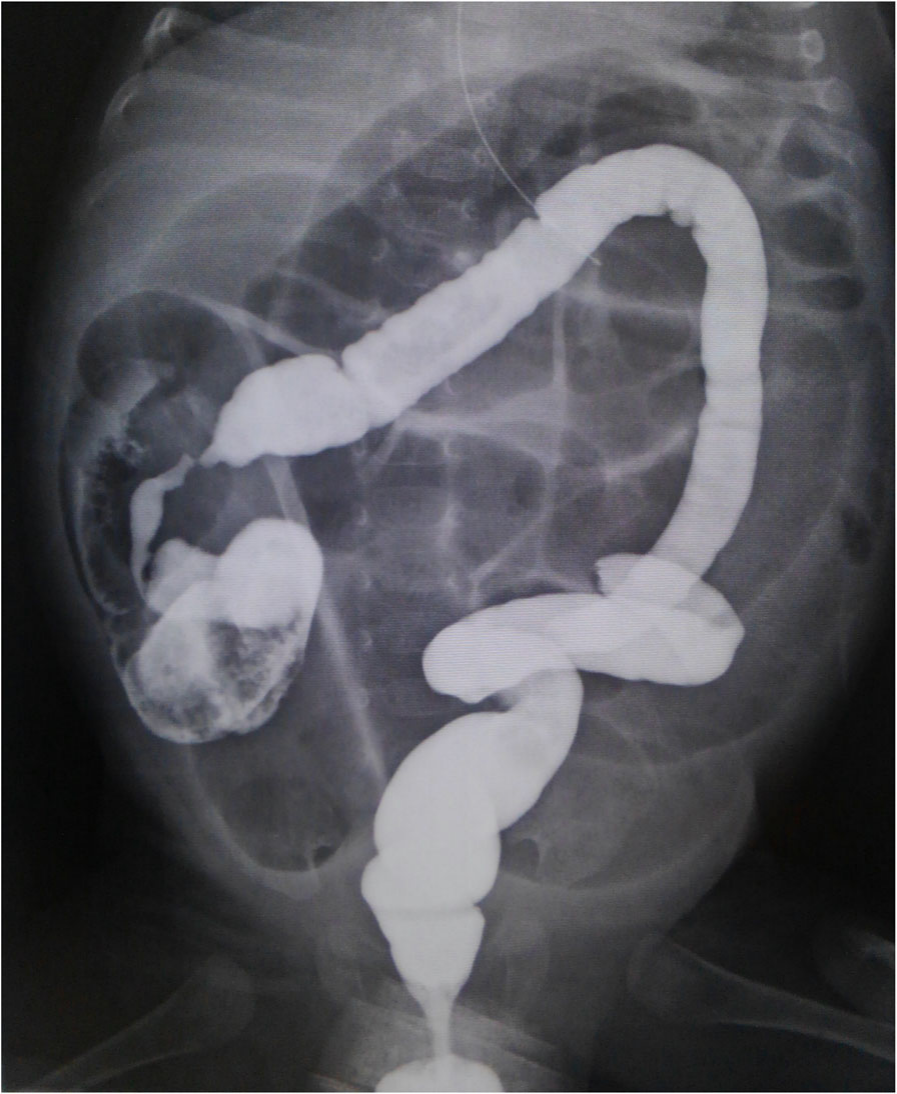
Contrast X-ray of the colon on day 50. Beak signs of ascending to the transverse colon suggested colon stenosis

However, from his 30th day, he presented with increasing apnea and vomiting. Contrast gastric X-ray performed on the 40th day revealed gastroesophageal reflux (GER). Treatment of GER was added by dividing enteral feeding and medication. Nevertheless, his quantity of stool gradually decreased, and physical examination showed worsening abdominal distention. Simultaneously, his CRP and WBC levels were slightly elevated up to his 40th day at 2.56 mg/dL and 20,380/μL, respectively. Intravenous cefazolin (100 mg/kg/day) was administered, followed by cefaclor p.o. Abdominal radiography on his 50th day showed a significantly distended intestine with gas (Fig. 1), which urged us to perform contrast X-ray of the colon; the X-ray suggested stenosis of the ascending colon (Fig. 2). In order to decompress the distended bowel, we tried to insert a tube through the narrowed colon resulting in failure. Therefore we decided to perform emergent exploratory laparotomy.

**Fig. 3:**
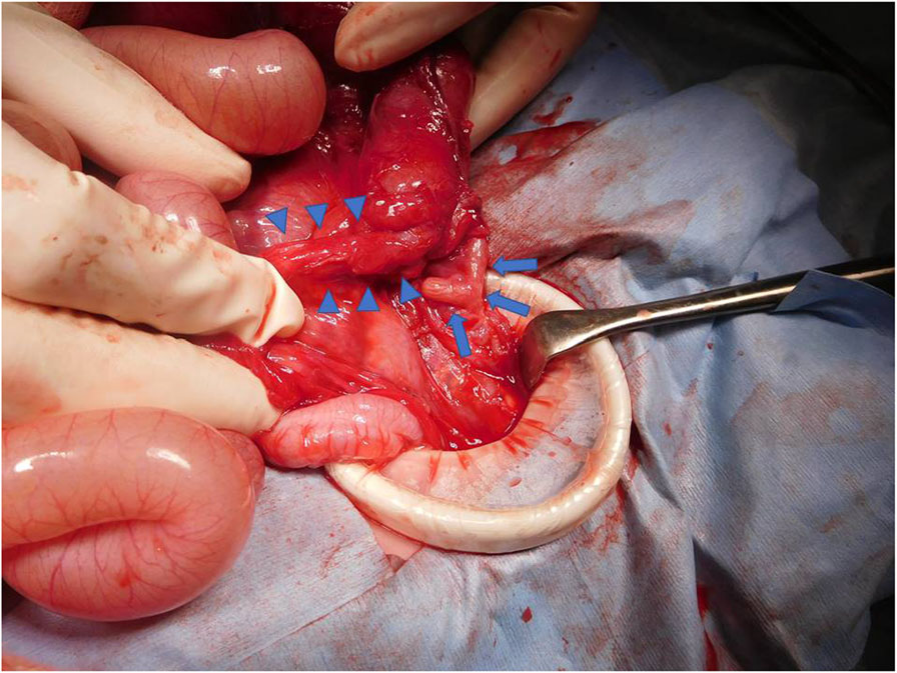
Macroscopic findings in the surgery. The appendix is adhered to the ascending colon (arrow). Part of the ascending colon including the adhered lesion shows stenosis (triangles)

Intraoperative findings included viable loops of bowel, and the terminal ileum remarkably distended in the right lower quadrant (Fig. 3). The appendix was adhered to the ascending colon by approximately 1 cm from the ileocecal junction and was found to be perforated at its root. This part of the colon was narrow and was not dilated by compression from the oral end, while the contents passed through smoothly. Ascites was slightly yellowish and was examined by a culture test, which turned out to be negative. No perforation in the colon or evident peritonitis was observed macroscopically. The ascending colon lesion including the appendix was resected by 3 cm, and then the ileum and remaining ascending colon were anastomosed end to end.

**Fig. 4:**
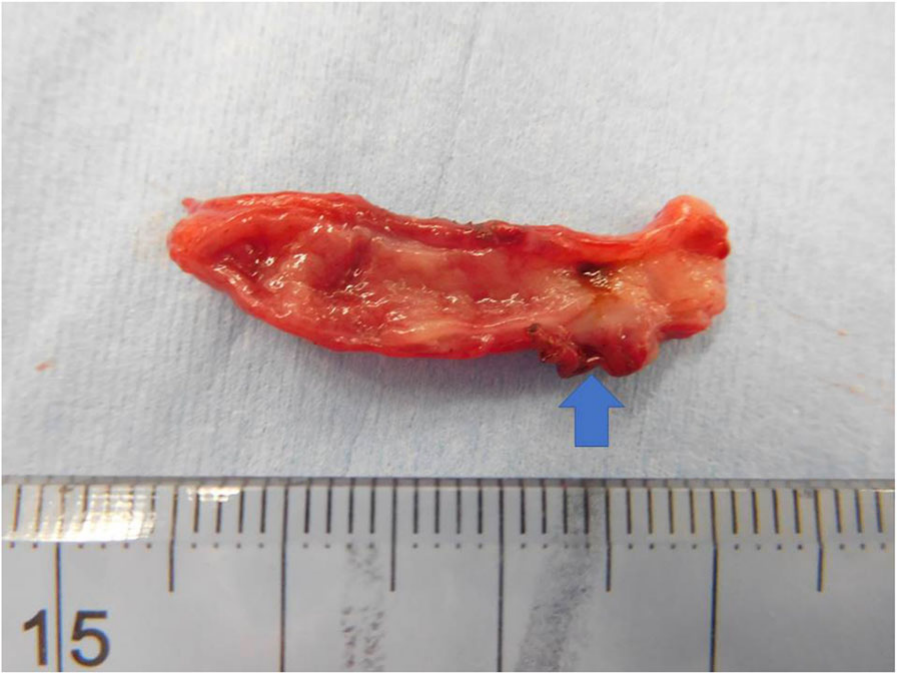
The resected appendix. The resected appendix shows perforation (arrow) in its root

**Fig. 5:**
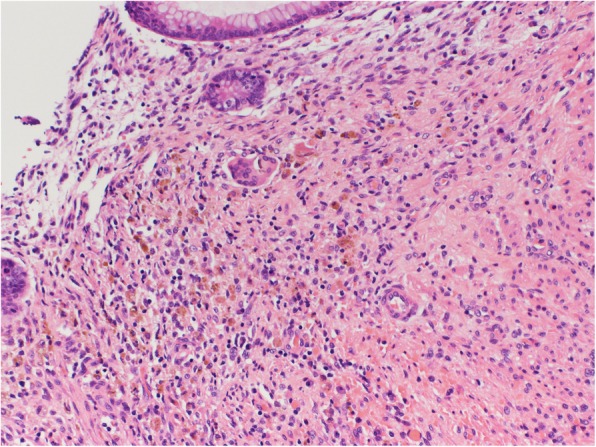
Appendix histopathology. High-power (400-fold magnification) view (hematoxylin and eosin staining) showing old hemorrhage, subtle calcification, and a few foreign body granulomas

The pathological findings were as follows: The appendix was perforated at a 2.5-mm diameter 5 mm from the root (Fig. 4). The mucosa showed lymphoid hypoplasia. Necrotic granulation tissue replaced the area around the perforation. Old hemorrhage, subtle calcification, and a few foreign body granulomas were found in the area; a finding that is compatible with several weeks of duration of the lesion (Fig. 5). By contrast, reconstruction of the luminal layers by fibrosis, suggesting months’ delay, was not observed.

**Fig. 6:**
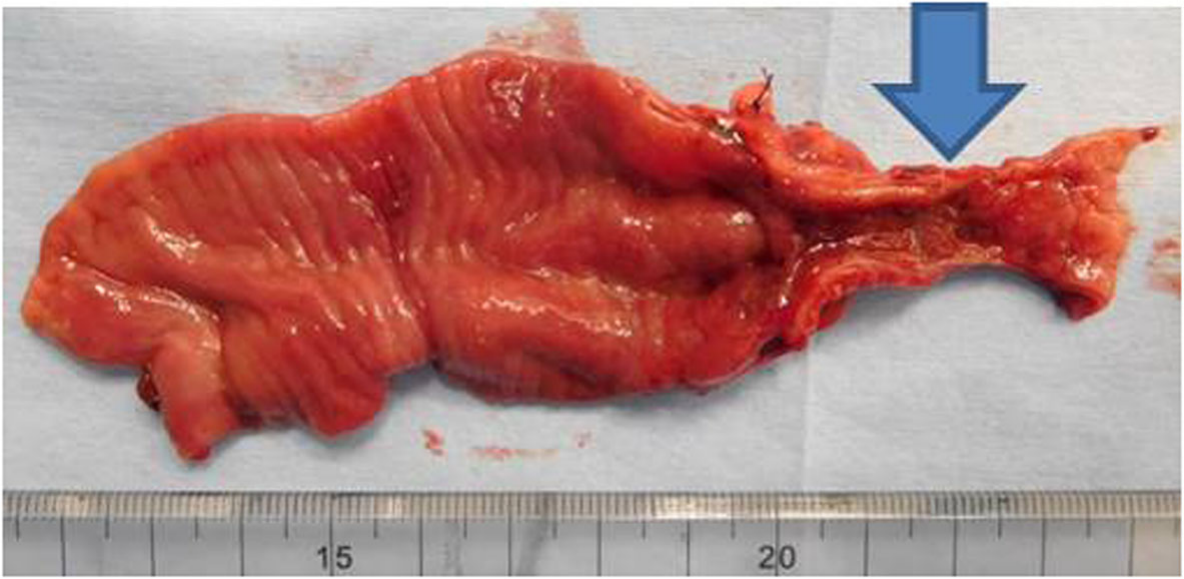
The resected ascending colon. Resected ascending colon showing stenosis (arrow) near the ileocecal valve

**Fig. 7:**
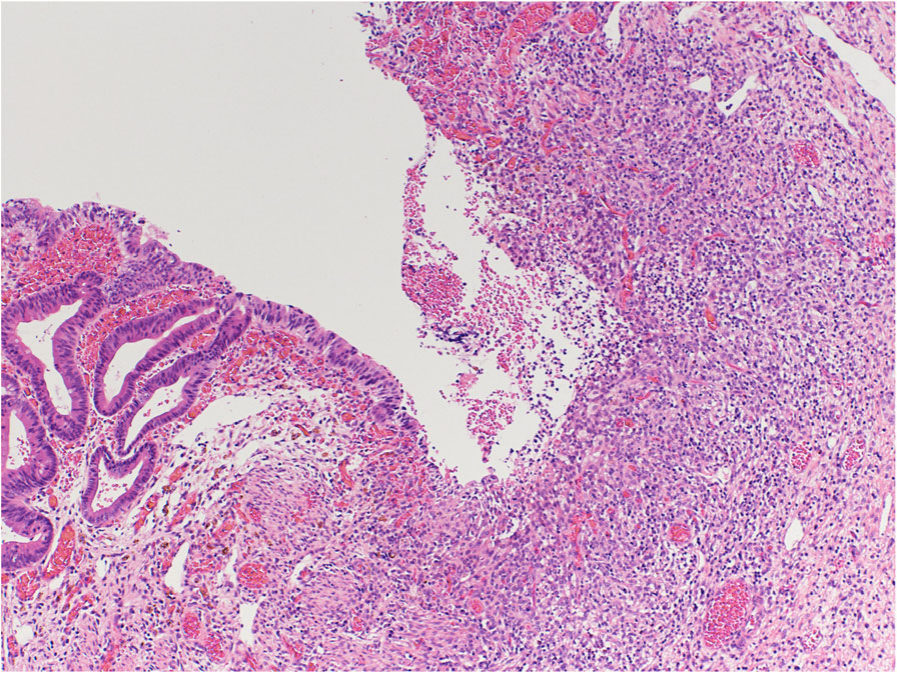
Ascending colon histopathology. High-power (400-fold magnification) view (hematoxylin and eosin staining) showing the erosion is a mucosal defect replaced by necrotic granulation, limited within the lamina muscularis mucosae and submucosa

Mucosal erosion of 18 mm in diameter was located on the ascending colon 7 mm from the ileocecal valve in the severely stenotic area (Fig. 6). The erosion was a mucosal defect replaced by necrotic granulation, limited within the lamina muscularis mucosae and submucosa. The submucosal granulation and fibrosis are compatible with several weeks of duration. (Fig. 7).

Postoperative recovery was good. Enteral feeding was resumed on postoperative day (POD) 4. He was tolerant of full feeding on POD 10. After surgery, he did not develop apnea and his GER treatment was completed. On POD 28, he weighed 3,040 g at the time of his discharge to home. Furthermore, 4 months after discharge, he developed adherent ileus and required surgical detachment. Currently, he is generally well and his neurological development is within the normal range.

## Discussion and conclusions

We experienced the preterm-boy case of colon stenosis due to NA. We believe that this is the first report of NA associated with colon stenosis among preterm babies.NA is a rare disease that occurs more frequently among preterm babies [1–3]. Additionally, few reports have been published on colon stenosis in preterm neonates [4, 5]. Nevertheless, these morbidities are worth attention.

NA presents high fatality, especially among preterm babies, and requires early intervention as soon as it is diagnosed [1]. Meanwhile, a paradox exists concerning NA; perforated cases have significantly higher survival rates in comparison with non-perforated cases. This is possibly due to delayed diagnosis and secondary inflammatory reactions, including sepsis [1]. In the case of our patient, although our surgical intervention was performed several weeks after the appendiceal perforation indicated by the pathological findings and his clinical course, our patient survived and thrived. Fortunately, in our case, the perforation of the appendix was covered by the ascending colon and this prevented the progression of peritonitis and possibly delayed the diagnosis or decision of surgical intervention. Anatomical proximity might have prompted such coverage. In addition, antibiotics were administered from day 7 to day 13, which might have partially treated NA and contributed to the prevention of peritonitis.

In spite of the necessity of early treatment, it is remarkably challenging to diagnose NA. No specific symptom or clinical test finding to diagnose NA has been reported. Symptoms of NA are generally unspecific, such as abdominal distension, vomiting, or anorexia [3].; therefore, it is imperative to keep NA in consideration as one of the differential diagnoses in newborns with gastrointestinal symptoms. Our patient presented vomiting and abdominal distension, and eventually reduced feces. His consecutive radiographs showed no signs of free air, which would suggest perforation of the intestinal tract, while his intestines extended remarkably implying that intestinal peristalsis was constrained. Although multiple blood cultures, urine cultures, and skin cultures turned out to be negative, the laboratory findings suggested persistent inflammations. CRP continued to be slightly elevated and most of the results were less than 1 mg/dL, except for 2.56 mg/dL on day 40. Given these findings, some ongoing inflammatory disorders in the intestinal tract might have been suspected.

In addition to the challenge of diagnosis, the onset of NA and perforation is also difficult to identify in a timely manner [3]. We believe that the neonate developed NA on day 7 when he had the initial abdominal symptoms and that the condition was partially treated with antibiotics. The histology suggested that the onset of the disease was several weeks before day 50, indicating day 7 as a possible time of onset. On the other hand, his clinical course included abdominal symptoms on day 7 and then on day 30. This course can lead to an alternative hypothesis that he suffered from NA from day 30. The question is whether the development of NA was on day 7 or 30. First, it would take a few weeks to several weeks from onset to develop colon stenosis, leading to symptoms such as stool reduction. Furthermore, from day 30 on, his vital signs were stable without treatment of NA. Due to the duration from the onset of NA to the subsequent colon stenosis and the relatively stable conditions that he experienced, we are inclined to assume the onset of NA to be earlier than day 30 and suspect day 7. Nonetheless, the histological findings of fibrosis in the appendix, suggesting passage for several weeks, are relevant to both of the hypotheses; as a result, we could not conclusively determine which day was the actual onset.

Several hypotheses have been proposed concerning the etiology of NA. They include a limited form of necrotizing enterocolitis (NEC), association with Hirschsprung disease, and colon obstruction due to meconium ileus [6]. NA is difficult to distinguish from NEC due to its nonspecific clinical presentations, especially among preterm babies. The relationship between NA and NEC is controversial; in this case, we could not conclude a direct attribution of NEC to its cause. However, our patient was not significantly predisposed to NEC [4]. Although he was born with low birth weight and relatively low gestational age, he did not require intubation or demonstrate a small size for his gestational age and did not exhibit premature rupture of membranes, sepsis, or hypotension. Furthermore, he was delivered by cesarean section, which has been controversially indicated as a protective effect on NEC [6].

According to our literature review, comorbidities of NA are scarcely reported. We believe that he first developed NA which resulted in the appendiceal perforation and his ascending colon covered its perforated site, which in turn gradually narrowed due to inflammatory reaction. The fibrosis in the appendix suggested the several weeks’ passage from the onset of NA and implied precedence of the appendiceal lesion to the colon. On the other hand, we cannot rule out the possibility that the primary lesion located in the ascending colon may have preceded and affected the appendicitis. If the colon lesion had occurred prior to NA, the possible etiologies of the primary colon lesion would have included NEC, congenital stenosis, and cytomegalovirus enterocolitis [4, 5]. If NEC had affected his ascending colon first, the consequent stenosis would have elevated inner pressure, which could have been the cause of appendiceal perforation; however, it is less likely that the perforated site of the appendix adhered to the very same locations as the NEC lesion. Epidemiologically, it is also less likely to have primary or congenital colon stenosis in the part of his colon which the appendix touched. Pathological findings revealed no evidence of cytomegalovirus infection.

Finally, regarding the management of preterm babies with apnea, it is worth revisiting that GER and apnea are caused secondarily by certain gastrointestinal events. In our case, his clinical symptoms first suggested GER, which is a common comorbidity during the management of preterm babies, and we also diagnosed GER based on the upper gastric contrast X-ray. Feeding was administered every 2 h, while his total water intake remained unchanged at 160 mL/kg/day. Additionally, the administration of the Chinese herb Rikkunshito, which prompts gastric clearance, was added. However, the progression of abdominal distension indicated possible obstructive or functional causes in his intestine and colon. Our patient showed persistent apnea presumably due to GER before surgery, and his apnea was completely cured after the postoperative extubation.

In conclusion, NA remains a challenging disease, with colon stenosis representing a rare complication. Their nonspecific symptoms present similarly in preterm babies. Exploratory laparotomy is useful for the diagnosis and treatment of both NA and colon stenosis when symptoms are worsening.

## Data Availability

Data sharing is not applicable to this article as no datasets were generated or analyzed during the current study.

## Abbreviations

CPAP: continuous positive airway pressure
CRP: C-reactive protein
GER: gastroesophageal reflux
NA: Neonatal appendicitis
NEC: necrotizing enterocolitis
NICU: neonatal intensive care unit
POD: postoperative day
WBC: white blood cell
